# Cecocolic Intussusception in Adult Caused by Acute Appendicitis

**DOI:** 10.1155/2014/108327

**Published:** 2014-02-19

**Authors:** Jeonghyun Kang, Kang Young Lee, Seung-Kook Sohn

**Affiliations:** Department of Surgery, Gangnam Severance Hospital, Yonsei University College of Medicine, 211 Eonju-ro, Gangnam-gu, Seoul 135-720, Republic of Korea

## Abstract

Intussusception in adult is rare. The etiology is different from that of childhood. The most common cause of intussusception in adult is known as malignancy. When dealing with adult intussusception, surgical resection is usually warranted for correct diagnosis and proper treatment. This is a case report of cecocolic intussusception caused by an acute appendicitis in adult. The causes of cecocolic intussusception were reported as appendiceal adenocarcinoma, appendiceal mucocele, appendiceal adenoma, or idiopathic. Although this patient underwent laparoscopic right hemicolectomy under suspicion of malignancy at cecum base, final pathologic diagnosis revealed only acute appendicitis. Thus, the present case emphasizes the importance of prior thorough examinations including colonoscopy when we encounter this rare kind of intussusception in adult.

## 1. Introduction

Intussusception occurs when one segment of bowel and associated mesentery invaginated into an adjacent segment [1]. Intussusception is primarily a childhood disease. Adult intussusception is a rare clinical entity accounting for 5% of all intussusceptions [1]. It was reported that 63% of the adult intussusception was related to the tumor [2]. Because of the high risk of malignancy, definitive surgical resection is the recommended treatment in nearly all cases in adult intussusception [2].

Among various kinds of intussusception in adult, cecocolic intussusception was rare. Although appendiceal adenocarcinoma, adenoma, or mucocele could cause cecocolic intussusception, acute appendicitis was rarely reported as a leading cause of cecocolic intussusception. We report a case of cecocolic intussusception caused by an acute appendicitis treated by laparoscopic right hemicolectomy.

## 2. Case Presentation

**Figure 1:**
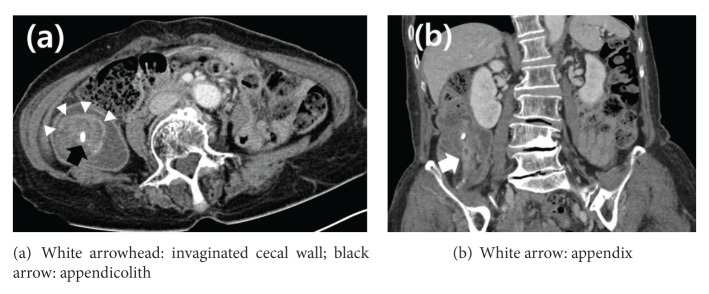
Abdominal computed tomography. Transverse (a) and coronal (b) views revealed invagination of the caecum and the appendix into the lumen of the ascending colon, as well as mural thickening and enhancement of the dilated appendix (9.7 mm) with a few appendicoliths.

In February, 2013, a 73-year-old woman presented to our emergency department with right lower abdominal pain which began 2 days prior to admission. She had no relevant medical or surgical history. Pain was a maximum of 5 on the visual analogue scale. Routine blood tests showed mild elevated C-reactive protein and white blood cell counts were within the normal range. Abdominal computed tomography (CT) scan revealed invagination of the cecum and appendix into the lumen of the ascending colon. Mural thickening and enhancement of the dilated appendix up to 1 cm with a few appendicoliths in the invaginated large bowel were also noted (Figure 1).

**Figure 2:**
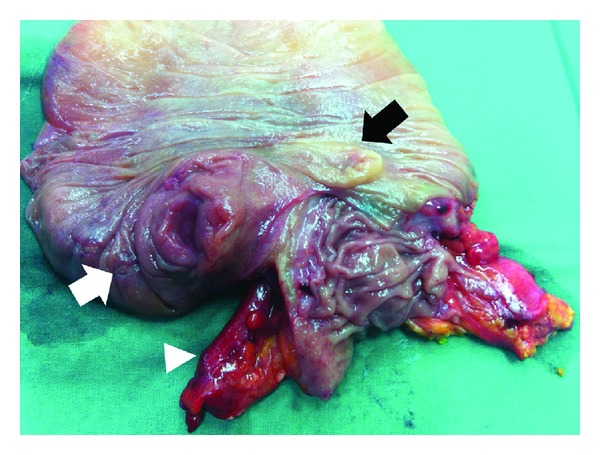
Resected specimen showing a hard, mass-like lesion in the cecum base (white arrow: mass-like lesion; black arrow: ileocecal valve; white arrowhead: appendix).

The patient underwent laparoscopic surgical resection. Inspecting the abdomen after general anaesthesia, the intussusception was already spontaneously resolved. The appendix showed only mild inflammation with no gangrenous change or perforation. It was unclear whether the cause of appendicitis was primary obstruction of the appendix lumen by appendicoliths or secondary spreading of adjacent inflammation induced by invagination. On thorough laparoscopic examination, a nonspecific thickening of the cecal wall near the appendix orifice was palpated by laparoscopic instruments. It was impossible to exclude the possibility of a hidden malignancy of the cecum. Thus, laparoscopic right hemicolectomy was performed. Although mural thickening around the appendix orifice was evident in the resected specimen (Figure 2), histological examination confirmed acute nonspecific suppurative inflammation in the appendix and cecum base. The patient's recovery was uneventful.

## 3. Discussion

Intussusception is a major cause of intestinal obstruction in children. However, it is rare in adults, representing only 5% of all intussusceptions [1]. Although it was reported that cecocolic intussusception was one of the most common types of intussusceptions in Western Nigeria and other parts of Africa [3], there have been only limited reports of cecocolic intussusception in other areas. The cause of cecocolic intussusception is typically appendiceal adenocarcinoma, appendiceal mucocele, appendiceal adenoma, or idiopathic [4–8]. To the best of our knowledge, cecocolic intussusception due to acute appendicitis has been rarely reported in the English literature.

Surgical intervention is strongly recommended in adult intussusception because the majority of cases of intussusception in adults are caused by malignancy. There is some debate regarding the need for reduction procedures. Theoretically reduction prior to resection could minimize the range of resection. Nevertheless, resection without reduction has been regarded as a more favourable procedure because reduction is not always easy, and the reduction process could cause tumour spillage. Thus, surgical resection is warranted for diagnosis and treatment in cases of adult intussusception.

In this case, we performed a laparoscopic right hemicolectomy because we could not eliminate the possibility of a malignancy of the base of the cecum owing to a hard, mass-like lesion and considering the patient's old age. Although right hemicolectomy is regarded as a viable option to prevent recurrence in adult ileocolic intussusception [9], final pathology did not reveal a malignancy. It was reported that the limited information with preoperative suspicion for malignancy resulted in excessive resection [10].

Lee et al. reported a case where cecocolic intussusception was reduced by air inflation during colonoscopy. Colonoscopic biopsy revealed adenocarcinoma of the appendix, and the patient underwent right hemicolectomy for treatment of appendiceal adenocarcinoma [5]. Interestingly, Tominaga et al. reported a 23-year-old female who underwent successful endoscopic reduction of idiopathic cecocolic intussusception without surgery [4]. Considering the role of colonoscopic examination in the above cases, prior examination before surgical intervention could have been beneficial in deciding the best surgical treatment option for our case.

In conclusion, our case demonstrated that, in the management of cecocolic intussusception in adults, an accurate diagnosis should be made to avoid unnecessary resection. When possible, the physician should consider thorough examinations including colonoscopy to investigate the pathologic leading point of intussusception before surgical intervention.
